# The influence of lifestyle habits on quality of life in patients with established rheumatoid arthritis—A constant balancing between ideality and reality

**DOI:** 10.3402/qhw.v11.30534

**Published:** 2016-05-10

**Authors:** Karina Malm, Ann Bremander, Barbro Arvidsson, Maria L. E. Andersson, Stefan Bergman, Ingrid Larsson

**Affiliations:** 1 Department of Clinical Sciences, Section of Rheumatology, Lund University, Lund, Sweden; 2 Spenshult Research and Development Center, Halmstad, Sweden; 3 Rheumatology, Capio Movement, Halmstad, Sweden; 4 School of Business, Engineering and Science, Halmstad University, Halmstad, Sweden; 5 School of Health and Welfare, Halmstad University, Halmstad, Sweden; 6 Primary Health Care Unit, Department of Public Health and Community Medicine, Institute of Medicine, The Sahlgrenska Academy, University of Gothenburg, Gothenburg, Sweden

**Keywords:** Lifestyle habits, quality of life, qualitative content analysis, rheumatoid arthritis

## Abstract

**Introduction:**

Rheumatoid arthritis (RA) is a chronic, inflammatory, and systemic disease with symptoms that limit activities and affect quality of life. RA is associated with an increased risk of developing comorbidities, some of which are also known to be associated with lifestyle habits such as physical activity, diet, smoking, and alcohol. There has been an augmented focus on the implementation and maintenance of healthy lifestyle habits even for patients with RA in the past decade, but little is known about the link between patients’ experiences of lifestyle habits and quality of life. The aim of the study was thus to describe and explore how patients with established RA experience the influence of lifestyle habits on quality of life.

**Methods:**

The study had a descriptive and explorative design, based on qualitative content analysis. Strategic sampling was used in order to achieve variations in experiences. Twenty-two patients with RA (14 women and 8 men) from 30 to 84 years old, with a disease duration ranging from 8 to 23 years, were interviewed.

**Results:**

The analysis of the influence of lifestyle habits on quality of life resulted in the theme *balancing between ideality and reality*. Three categories emerged about how lifestyle habits influenced quality of life by *limitations* (including insufficiency and adaptation), *self-regulation* (including guilt and motivation), and *companionship* (including belonging and pleasure).

**Conclusions:**

Quality of life for patients with established RA was influenced by the balance between ideality and reality in the lifestyle habits: physical activity, diet, smoking, and alcohol. This is important new knowledge for health professionals when discussing lifestyle habits with RA patients.

Rheumatoid arthritis (RA) is a chronic, inflammatory, and systemic disease of unknown etiology with a prevalence of 0.5–1.0% in the western world. The average age at onset is the mid-50s, and the female to male ratio is three to one (Englund et al., 2010; Scott, Wolfe, & Huizinga, 2010). Because the disease is chronic, the patients have to live with lifelong effects from their RA and early diagnose and treatment will improve the long-time prognosis (Monti, Montecucco, Bugatti, & Caporali, 2015). Based on disease duration, the term early RA is generally defined as having less than 2 years of disease duration, whereas a longer disease duration is referred to as established RA (Conaghan, Green, & Emery, 1999).

Pain, physical disability, fatigue, and sleep disturbances are some of the most pronounced symptoms in patients with a more severe disease progression or with a longer disease duration, resulting in activity limitations seriously affecting health-related quality of life (Uhlig, Moe, & Kvien, 2014). RA is also associated with an increased risk of developing comorbidities (Dougados et al., 2014; Turesson & Matteson, 2007), some of which are known to be associated with lifestyle habits such as a sedentary lifestyle, unhealthy diets, smoking, and alcohol (Beaglehole et al., 2011).

Studies have shown that patients with RA do not reach health-enhancing levels of physical activity which affects their cardiovascular health (Sokka et al., 2008). Diet and obesity are closely linked, and obesity is associated with worse disease severity and a larger number of comorbidities in patients with RA (Ajeganova, Andersson, & Hafstrom, 2013). Patients with cachexia (loss of muscle mass and strength and concomitant increase in fat mass) also have an increased risk of cardiovascular diseases (Elkan, Hakansson, Frostegard, Cederholm, & Hafstrom, 2009).

Substance use, such as smoking and alcohol, has disease-specific effects, besides well-known general health effects. Smoking, which is the known environmental factor that has the greatest significance for the development of RA, is associated with a worse prognosis and is also a negative factor in terms of response to therapy (Soderlin, Petersson, Bergman, Svensson, & BARFOT study group, 2011; Soderlin, Petersson, & Geborek, 2012). A moderate consumption of alcohol may have a positive effect on disease activity, and prognosis and may decrease the risk of developing RA (Maxwell, Gowers, Moore, & Wilson, 2010). The importance of a healthy lifestyle in addition to antirheumatic drugs and other treatment modalities is well known for patients with RA, and health professionals are encouraged to prioritize discussions on lifestyles habits when supporting patients (Dean & Gormsen Hansen, 2012).

There is limited knowledge about the impact of lifestyle habits such as physical activity, diet, smoking, and alcohol on quality of life. In the general population, physical activity has been reported to be positively associated with quality of life (Bize, Johnson, & Plotnikoff, 2007; Tessier et al., 2007). Both a vegetarian diet and a Mediterranean diet may improve quality of life in patients with established RA (Li & Micheletti, 2011; Skoldstam, Hagfors, & Johansson, 2003). Smoking is negatively associated with quality of life and the association is related to the number of cigarettes smoked, whereas smoking cessation is associated with an improved quality of life (Goldenberg, Danovitch, & IsHak, 2014). There are studies reporting moderate alcohol use having a beneficial effect on quality of life, but there is no consensus whether it is the alcohol or the social context affecting the quality of life (Bergman, Symeonidou, Andersson, & Soderlin, 2013; Kim et al., 2013).

The aim of the study was thus to describe and explore how patients with established RA experience the influence of lifestyle habits on quality of life.

## Method

### Design

The study had a descriptive and explorative design, based on qualitative content analysis, with an inductive approach. Qualitative content analysis is a method that provides a systematic way of making valid inferences from verbal or written data in order to describe a specific phenomenon (Krippendorff, 2013). The method is often used in healthcare research (Graneheim & Lundman, 2004). Although described as lacking a solid theoretical background (Krippendorff, 2013), qualitative content analysis is useful for providing knowledge and understanding of a studied phenomenon and for analyzing a person's experiences, reflections, and attitudes (Krippendorff, 2013). The intention is to describe variations by identifying differences and similarities in the content, both the manifest content (describing what the text says) and the latent content (exploring the interpreted meaning) (Graneheim & Lundman, 2004).

### Participants

A total of 2800 adult patients over 18 years old were enrolled in the BARFOT (Better anti-Rheumatic FarmacOTherapy) study from 1992 to 2006; a multicenter longitudinal observational study of patients with early RA in southern Sweden. All patients in the BARFOT study (*n*=2100) received a postal questionnaire in 2010 concerning lifestyle factors, including physical activity, diet, smoking, and alcohol habits. The interviews for the present study were conducted with 22 of them, a common number of informants in qualitative content analysis (Graneheim & Lundman, 2004; Mason, 2010). The informants were selected by using a strategic sampling procedure in order to get variation in physical function (Health Assessment Questionnaire, HAQ), quality of life (EuroQol5D, EQ-5D), sex, age, marital status, education, disease duration, employment, and all six BARFOT centers which cover both urban and rural patient referral areas (Table I).

**Table I:** Characteristics of the participants (*N*=22).

Participant (P)	BARFOT Clinic	HAQ	EQ-5D	Sex	Age	RA-duration (years)	Married/Co-habiting	Education	Employment
1	3	1.13	0.59	Female	63	14	Yes	Compulsory comprehensive school	Retirement- disease
2	3	0.88	0.73	Female	52	16	No	Upper secondary school	Retirement- disease
3	3	1.38	0.73	Male	68	20	Yes	Upper secondary school	Retirement
4	5	1.38	0.73	Female	65	20	Yes	Undergraduate studies	Part time
5	6	0.63	0.73	Male	66	17	Yes	Compulsory comprehensive school	Retirement
6	6	1.13	0.80	Female	77	8	Yes	Compulsory comprehensive school	Retirement
7	4	0.63	0.73	Female	63	21	No	Undergraduate studies	Full time
8	2	0.75	0.66	Male	65	22	No	Upper secondary school	Retirement
9	4	0.38	0.66	Female	65	11	Yes	Upper secondary school	Retirement
10	4	1.0	0.52	Male	63	18	Yes	Compulsory comprehensive school	Part time
11	3	0.50	0.73	Female	64	22	Yes	Upper secondary school	Sick leave
12	1	1.0	0.52	Female	51	12	Yes	Upper secondary school	Sick leave
13	1	0	1.00	Male	84	18	Yes	Compulsory comprehensive school	Retirement
14	1	0	0.85	Female	77	17	Yes	Upper secondary school	Retirement
15	1	0	1.00	Male	38	19	No	Upper secondary school	Full time
16	5	0	0.80	Male	68	19	Yes	Compulsory comprehensive school	Retirement
17	2	0	0.80	Female	30	10	Yes	Undergraduate studies	Part time
18	2	0.50	0.66	Male	74	14	No	Compulsory comprehensive school	Retirement
19	2	0.88	0.69	Female	58	23	No	Upper secondary school	Sick leave
20	3	0	0.80	Female	37	14	No	Undergraduate studies	Full time
21	3	0	0.85	Female	32	9	Yes	Undergraduate studies	Full time
22	3	0	1.00	Female	40	13	Yes	Upper secondary school	Full time

### Data collection

Data collection took place at the participant‘s BARFOT clinic in a private room between May 2014 and June 2015. The interview started with the main author (KM) clarifying the aim of the study. All interviews were initiated with an open-ended question: How do your lifestyle habits influence your quality of life? The focus was on the lifestyle habits: physical activity, diet, smoking, and alcohol. In order to encourage the patients to think more profoundly about the question and to obtain in-depth data, follow-up questions such as ”Please, can you tell me more about …?”, or ”How do you mean? …” were asked. Two pilot interviews were conducted to test the opening question and these interviews were included in the study because no amendment was required. Each interview lasted 30–70 min and was audio recorded.

### Data analysis

Qualitative content analysis was used, and both the manifest and latent content were analyzed in accordance with Graneheim and Lundman (2004). The analysis was performed by the authors KM, BA and IL. The authors AB, MA, and SB reviewed the analysis and discussions were held until consensus was reached. The main author (KM) transcribed the interviews verbatim. The interviews were read through several times in order to gain familiarity, and meaning units related to the aim of the study were identified, resulting in 526 meaning units. These were condensed to shorten the text while retaining the content. The condensed meaning units were abstracted to codes that were compared, based on similarities and differences, after which they were grouped into seven subcategories and three categories on a manifest level. The content of these categories was brought together and an overall theme emerged, which expressed the latent meaning of the content.

### Ethical considerations

The Regional Ethical Review Board at Lund University, Sweden, approved the study LU 2014/146. The participants received a letter regarding the aim of the study, its design, and the voluntary nature of participation and gave their informed consent. The participants were guaranteed confidentially and informed that they could withdraw at any time without giving an explanation and without any consequences for future care. The study was carried out in accordance with the ethical principles of the Declaration of Helsinki 2013 (WMA, 2013).

## Findings

### Balancing between ideality and reality

The quality of life for participants with established RA was influenced by the balance between ideality and reality in the performance of lifestyle habits: physical activity, diet, smoking, and alcohol. These could be described in terms of limitation, self-regulation, and companionship, which influenced their experience of their quality of life, whilst they constantly balanced between ideality and reality (Table II). Their experiences of these were individual and diverse, and their quality of life was influenced both positively and negatively. These habits were performed automatically, without continually analyzing the potential effect on their quality of life.

**Table II:** The themes, categories, and subcategories reflecting the influence of lifestyle habits on the quality of life of patients with rheumatoid arthritis.

Theme	Balancing between ideality and reality		
Categories	Limitation	Self-regulation	Companionship
Subcategories	Insufficiency	Guilt	Belonging
	Adaptation	Motivation	Pleasure

### Limitation

The category limitation included the subcategories insufficiency and adaptation. Limitations in terms of insufficiency and adaptation in the performance of their lifestyle habits affected the balance between ideality and reality and thus influenced the quality of life.

The participants described a limitation in *physical activity* in everyday situations because of flashes of pain in their joints, stiffness, and fatigue affecting quality of life. They were not able to walk in some environments and to perform some activities, which led to a sense of *insufficiency* affecting quality of life. They expressed an *insufficiency* in terms of trying certain activities, of being physically active, of a fear of falling, and not being able to manage the situation. The participants indicated how they used different types of strategies and *adaptations* to overcome their limitations and to be able to become, or to remain physically active. They talked of different types of adapted training equipment and technical aids to facilitate walking, which influenced quality of life.

I know that I'm very limited. I can't, I can't go out in the woods because I can't walk on such uneven ground, I just can't (P 4).

In relation to *diet*, the participants expressed a limitation with feelings of *insufficiency* by having a lack of knowledge about how diet affects their health and thus their quality of life. There was a fear of having an unhealthy diet and of gaining weight. The participants *adapted* themselves by active decisions to avoid food that could cause them to gain weight. They adapted their diet after the family's needs even if it affected their health and therefore their quality of life.

Now when I live alone and can decide that this food makes me feel good, it works for me, so my quality of life in terms of food has become much better (P 2).

Participants who *smoked* felt a limitation through *insufficiency*. They knew about the harmful effects of smoking and expressed a feeling of insufficiency and a fear of not being able to stop smoking. There were participants who had *adapted* themselves to the recommendations and stopped smoking.

I have to stop smoking now and I've had, I've tried several times before but it hasn't worked properly (P 9).

The participants spoke of a limitation through an *insufficient* knowledge about the effects of *alcohol* on their health and thus quality of life. They expressed a concern about drinking alcohol because of the harmful effects. There were also informants who had *adapted* their use of alcohol after disease onset and drug treatment, even if it resulted in a restriction on their lives and affected their quality of life.

Too much would most likely be negative for …. for my quality of life, but a limited amount of alcohol would, I think, raise my quality of life (P 20).

### Self-regulation

The category self-regulation included the subcategories guilt and motivation. Self-regulation in form of guilt and motivation in the performance of their lifestyle habits were factors that influenced the quality of life.

The self-regulation in *physical activity* was affected by a feeling of *guilt* about being physically inactive. The participants expressed a desire and *motivation* to be physically active, in spite of not being physically able to, because they saw an opportunity to improve their quality of life. Their motivation to perform physical activity at home, at work, and during leisure time was based on individual experiences.

Well, without physical activity, there is no quality of life either. Then you will just be on the couch, and that is the worst thing you can do, so it hangs together (P 19).

The participants experienced that their self-regulation was affected by *guilt* if their *diet* was unbalanced. Opinions of family and friends concerning what diet would be the best for the participants made them feel guilty. The opposite also emerged and there were participants who expressed a *motivation* to change to a healthy diet and they experienced an effect on their quality of life.

But, I don't perhaps eat enough fish, it may be so, but we have it once a week at least (P 5).

The *smoking* induced a sense of *guilt* because smoking affected their health and RA treatment. The participants described that self-regulation was driven by a *motivation* and an opportunity to influence their health and thus their quality of life by stopping smoking.

Perhaps because it could affect my RA, it's not so good and it's perhaps because of smoking that I got RA. But I never really did enough (P 9).

Self-regulation in relation to *alcohol* was described by the participants with varied emotions. Those who consumed alcohol, knowing that it was dangerous for their health in combination with their medication, felt *guilty*. It appeared that they were *motivated* to abstain from alcohol in order to achieve better quality of life, but that they also experienced an opportunity for good health and improved quality of life by drinking small amounts of alcohol.

I just will not feel better if I have a glass of wine. It doesn't really matter or so. And I have stopped partying because it did actually have a negative impact [on quality of life]. I often got more pain after a night out than if I didn't party (P 21).

### Companionship

The category companionship included the subcategories belonging and pleasure. Companionship in terms of belonging and pleasure in the performance of the lifestyle habits influenced the quality of life and was a part of the balancing between ideality and reality for patients with established RA.

The participants experienced that *physical activity* was based on a sense of *belonging*, a feeling of being in a companionship. The participants also expressed the opposite, a sense of loneliness if they were limited in carrying out physical activity. To be able to participate in activities was described as a source of *pleasure* with an impact on quality of life.

It feels great and you cycle a little and feel quite good and greet a few people (P 10).

Companionship in relation to *diet* was perceived as a sense of *belonging*. The participants described a sense of *pleasure* when eating together with family and friends that had a positive impact on their quality of life.

When one can cook something that tastes really good and everyone is satisfied. That's life quality (P 3).

The participants who *smoked* experienced a sense of *belonging* to the companionship of smokers. They describe a pleasure with the smoking and an influence on quality of life. A sense of *belonging* even emerged among those participants who did not smoke and a *pleasure* from not being exposed to smoking.

There's no one in my neighborhood who smokes, not anyone, no neighbors or anything. That's good (P 4).

Companionship also emerged in relation to *alcohol* ****, where the participants expressed that alcohol created a sense of *belonging*, with an impact on quality of life. The participants experienced *pleasure* and enjoyed eating and drinking in a social companionship.

It can be very cosy when you're together in a group or when you get a really good liqueur with a cup of coffee and a piece of cake or something (laughter) … then it's fine, then I think it's nice and cosy (P 1).

## Discussion

This study explored the influence of lifestyle habits on quality of life in patients with established RA. The result showed that the experience of quality of life depended on a constant balancing of performed lifestyle habits between the ideal situation (ideality) and the actual situation (reality), including limitation, self-regulation, and companionship. Lifestyle habits, such as physical activity, diet, and substance use, can be referred to as health-related habits and are internalized health determinants carried out automatically. They may contribute to positive health or to ill health and are targets for health improvement (Salvador-Carulla et al., 2013).

There has been an augmented focus on the implementation and maintenance of healthy lifestyle habits even for patients with RA in the last decade, but little is known of the link between patients’ experiences of lifestyle habits and quality of life. From earlier studies, we know that lifestyle habits, such as physical activity and diet, can be included in patients’ understanding of both the health concept and of the concept quality of life (Fagerlind, Ring, Brulde, Feltelius, & Lindblad, 2010).

For patients with RA, quality of life is considered as a composite of several dimensions of health consequences, including pain, fatigue, physical functioning, and social functioning (Kvien & Uhlig, 2005). This is in line with findings in the present study and previous research (Fagerlind et al., 2010). The findings indicate that living with established RA means a continuous balancing between ideality and reality. Patients with RA are vulnerable and relate individually to lifestyle habits and their impact on quality of life. There is an individual understanding of health and quality of life. They express a need to take active choices and to change priorities in everyday life and in lifestyle habits in order to enhance quality of life, which is consistent with previous findings by Thomsen et al. (2015). There is a constant balancing in everyday life and a need for adaptation in lifestyle habits. In order to maintain the balance in everyday life, with a fluctuating RA, patients try to regain control over their limitations by self-regulation (Flurey, Morris, Richards, Hughes, & Hewlett, 2014). Coping with arthritis is a balancing process, where patients redefine what they considered to be normal life throughout the disease (Gronning, Lomundal, Koksvik, & Steinsbekk, 2011).

In this study, patients with established RA experienced different limitations in relation to lifestyle habits (physical activity, diet, smoking, and alcohol). They expressed insufficiency, fear, and adaptation in everyday life and how it affected their quality of life. Limitations in relation to physical activity affect various activities in everyday life, due to lack of energy, presence of pain, and fear of joint damage (Van den Berg, De Boer, le Cessie, Breedveld, & Vliet Vlieland, 2007). The limitations can be influenced by biological factors, such as cartilage damage, joint degeneration, inflammation, swelling, and deconditioning (Keefe et al., 2002). It is known that pain and impaired function affect everyday activities in patients with early and longstanding RA (Malm, Bergman, Andersson, & Bremander, 2015; Thyberg, Dahlstrom, Bjork, Arvidsson, & Thyberg, 2012). Patients expressed an insufficiency about diet and special dietary recommendations to reduce RA symptoms. They state that these are hard to follow and sustain for a longer duration and they express limitations about cooking two dishes, one for the family and one for themselves (Ryden & Sydner, 2011). In this study, the patients who smoked expressed an insufficiency and fear of not being able to quit smoking, and they also said that they did not know how smoking affected the RA disease, which is in accordance with the findings of Aimer, Stamp, Stebbings, Valentino, et al. (2015), who described key barriers or limitations for smoking cessation. Patients are unaware of the relationship between smoking and RA, smoking is a distraction from pain and is used like a coping mechanism to handle the symptoms from the RA disease (Aimer, Stamp, Stebbings, Valentino, et al., 2015). The patients in the present study expressed a fear and an ambivalence in relation to alcohol and RA, which can be due to an association between alcohol and quality of life (Bergman et al., 2013).

The patients in this study described a self-regulation, including both guilt and motivation, in their way of handling or not being able to handle the lifestyle habits in order to gain quality of life. The findings showed that patients with established RA felt guilty when being physically inactive and quality of life was affected in a negative way. This result differs from a comparative study reporting that generalized feelings of guilt and shame are not major issues for patients with established RA and that they do not experience more feelings of guilt than their peers without RA. Guilt and shame are associated with demographic and psychosocial characteristics and not with physical functions and pain (Ten Klooster et al., 2014). The patients expressed a motivation to be physically active in improving quality of life. Lifestyle interventions are important for enhancing quality of life and motivation for physical activity among patients with RA (Knittle et al., 2015). The patients spoke of quality of life being influenced both by guilt and motivation in relation to physical activity, a healthy diet, smoking cessation, and by having a low alcohol consumption. Implementation of healthier dietary habits is difficult. Patients with RA are sometimes recommended a dietary change but find it difficult to achieve it outside their home. Their motivation for sustained dietary changes is individual, and the patients’ view on dietary changes is often that they are a temporary change during a specific period of time and not a permanent change of lifestyle habits. This supports the need for and the importance of sustainable lifestyle interventions in the context of dietary change (Ryden & Sydner, 2011) and RA-specific smoking cessation intervention in order to influence quality of life, and to avoid comorbidities and less effective therapy response (Aimer, Stamp, Stebbings, Cameron, et al., 2015).

The present study shows the importance of being a part of a companionship in order to experience quality of life in relation to lifestyle habits. Patients’ experiences of how lifestyle habits influenced quality of life in the social context of interacting with others included a sense of belonging and pleasure, which is in line with an earlier study stating that being physical active was an opportunity to experience a sense of companionship (Loeppenthin et al., 2014). It also included a duality, however, where an improved quality of life could be related to “unhealthy” behavior, such as physical inactivity, unhealthy eating, smoking, and drinking in a positive social context. Companionship sometimes contributed to physical inactivity, where patients with RA often spent time together with family and friends eating or just talking instead of being physically active (Thomsen et al., 2015). It has also been maintained that inactivity affects companionship negatively, when patients have high levels of pain and fatigue and difficulties leaving their homes, and do not have the energy to participate in valued social activities (Feldthusen, Bjork, Forsblad-d'Elia, & Mannerkorpi, 2013; Thomsen et al., 2015).

### Methodological consideration

Trustworthiness in a qualitative study should be based on the four criteria: credibility, dependability, confirmability, and transferability (Graneheim & Lundman, 2004; Polit & Tanto Beck, 2012). In order to strengthen the credibility, a strategic selection of participants was chosen. Credibility was also supported by the fact that two pilot interviews and a total of 22 interviews were conducted and that no new content in the subcategories emerged after the 16th interview. Rich descriptions are not primarily based on the number of participants but are obtained by qualitative inquiry into the experiences of the phenomenon in question. The interview text was deemed rich and contained great variety. Dependability was strengthened by the interviews being performed with the same main question, and that the informants were encouraged to talk openly (Graneheim & Lundman, 2004; Polit & Tanto Beck, 2012). Confirmability was demonstrated because of the systematic treatment of data. As a researcher, it is important to reflect on one's own attitudes and pre-understanding of the phenomena in question. When analyzing the interviews KM read individually all the written interviews and thereafter discussed the findings with two of the other authors (IL and BA). In the final process, all the co-authors participated in the construction of the final findings but no member check was done. This process required a genuine openness, flexibility, reflection, and critical discussion within the research group. The chosen quotations reflecting the content of each category provides the reader with an opportunity to determine the confirmability of the study (Polit & Tanto Beck, 2012). Transferability means that the study identifies the phenomenon that is set out to study, that is, how patients with established RA experience the influence of lifestyle habits on health-related quality of life. The transferability is strengthened because the study was performed among a national sample of BARFOT-clinics in Sweden. The findings could be transferable to another context (Graneheim & Lundman, 2004; Polit & Tanto Beck, 2012). A limitation could be that all the participants were born in Sweden.

## Conclusion

Quality of life for patients with established RA was influenced by the balance between ideality and reality in the lifestyle habits: physical activity, diet, smoking, and alcohol. These lifestyle habits could be described in terms of limitation, self-regulation, and companionship, all influencing the perception of quality of life, with a constant balancing between ideality and reality. The patients experience a struggle to determine the right balance of lifestyle habits in order to enhance quality of life and to try to achieve a normal life, independent of the established RA. This is important new knowledge for health professionals when discussing lifestyle habits with RA patients.
